# Microbial synthesis of zinc oxide nanoparticles and their potential application as an antimicrobial agent and a feed supplement in animal industry: a review

**DOI:** 10.1186/s40104-019-0368-z

**Published:** 2019-07-09

**Authors:** Hidayat Mohd Yusof, Rosfarizan Mohamad, Uswatun Hasanah Zaidan, Nor’ Aini Abdul Rahman

**Affiliations:** 1 Department of Bioprocess Technology, Faculty of Biotechnology and Biomolecular Sciences, Universiti Putra Malaysia, 43400 Serdang, Selangor Malaysia; 2 Bioprocessing and Biomanufacturing Research Centre, Faculty of Biotechnology and Biomolecular Sciences, Universiti Putra Malaysia, 43400 Serdang, Selangor Malaysia; 3 Department of Biochemistry, Faculty of Biotechnology and Biomolecular Sciences, Universiti Putra Malaysia, 43400 Serdang, Selangor Malaysia

**Keywords:** Animals, Antimicrobial, Feed supplement, Microbial synthesis, Nanotechnology, Zinc oxide nanoparticles

## Abstract

In recent years, zinc oxide nanoparticles (ZnO NPs) have gained tremendous attention attributed to their unique properties. Notably, evidence has shown that zinc is an important nutrient in living organisms. As such, both prokaryotes and eukaryotes including bacteria, fungi and yeast are exploited for the synthesis of ZnO NPs by using microbial cells or enzyme, protein and other biomolecules compounds in either an intracellular or extracellular route. ZnO NPs exhibit antimicrobial properties, however, the properties of nanoparticles (NPs) are depended upon on their size and shape, which make them specific for various applications. Nevertheless, the desired size and shape of NPs can be obtained through the optimization process of microbes mediated synthesis by manipulating their reaction conditions. It should be noted that ZnO NPs are synthesized by various chemical and physical methods. Nonetheless, these methods are expensive and not environmentally friendly. On that account, the microbes mediated synthesis of ZnO NPs have rapidly evolved recently where the microbes are cleaner, eco-friendly, non-toxic and biocompatible as the alternatives to chemical and physical practices. Moreover, zinc in the form of NPs is more effective than their bulk counterparts and thus, they have been explored for many potential applications including in animals industry. Notably, with the advent of multi-drug resistant strains, ZnO NPs have emerged as the potential antimicrobial agents. This is mainly due to their superior properties in combating a broad spectrum of pathogens. Moreover, zinc is known as an essential trace element for most of the biological function in the animal’s body. As such, the applications of ZnO NPs have been reported to significantly enhance the health and production of the farm animals. Thus, this paper reviews the biological synthesis of ZnO NPs by the microbes, the mechanisms of the biological synthesis, parameters for the optimization process and their potential application as an antimicrobial agent and feed supplement in the animal industry as well as their toxicological hazards on animals.

## Introduction

Over the last decade, nanotechnology has emerged as a technology that has revolutionized every field of applied science. The field of nanoparticles (NPs) is one of the avenues to nanotechnology that is associated with nanoscale materials with very small particles size ranging from 1 to 100 nm. NPs exhibit distinctive properties owing to their extremely small size and high surface area to volume ratio, which have attributed to the significant differences in the properties over their bulk counterparts [1]. In this regard, NPs have been integrated into various industries by providing innovative solutions.

There are various types of metal oxide including titanium dioxide (TiO_2_), indium (III) oxide (In_2_O_3_), zinc oxide (ZnO), tin (IV) oxide (SnO_2_) and silicon dioxide (SiO_2_), where ZnO is one of the abundantly produced metal oxides after SiO_2_ and TiO_2_ [2]. ZnO is an inorganic material that exhibits unique properties including semiconductor, a wide range of radiation absorption, piezoelectric, pyroelectric and possesses high catalytic activity [3]. In addition, ZnO has been listed as “Generally Recognized as Safe” (GRAS) by the US Food and Drug Administration (FDA 21CFR182.8991) [4] due to its non-toxic properties [5]. Consequently, this makes it safe to be used on human and animals. In recent years, there has been increased interest in zinc oxide nanoparticles (ZnO NPs). This is mainly due to their smallest particles size, which enhances their chemical reactivity. Consequently, this has extended the wide application of ZnO NPs in electronics, optics, biomedicine and agriculture [6–9].

Zinc are an important nutrient in living organisms [9–11]. Evidence has indicated that ZnO NPs have a great potential in biological applications, particularly as the antimicrobial agents [12, 13]. Moreover, numerous studies have been reported on the efficiency of ZnO NPs in inhibiting the growth of broad-spectrum of pathogens [14–16], which potentially could replace the conventional antibiotic. Furthermore, zinc is an important trace mineral that plays a vital role in many physiological functions in the body [9, 11, 17, 18]. As such, the integration of NPs in feed would increase the absorption and efficient use of zinc in the body, hence, result in improved health and productivity [19]. Moreover, evidence has indicated that ZnO NPs exhibit potential applications in the poultry and livestock industries, particularly as a feed supplement in the animal’s diet [9]. Numerous studies have been carried out to verify the potential use of ZnO NPs as dietary supplement in improving the growth performances [20–22], increase in the bioavailability of zinc [23, 24], enhancing the immune response [18, 25, 26], enhancing the antioxidative property [25, 27] and also improving the egg qualities and productions of layer chicken [24, 25, 28]. Nevertheless, to date, data on the use of ZnO NPs produced by microbial synthesis for the applications in animal feed has been scant.

Traditionally, ZnO NPs are synthesized using physical and chemical processes, which offer higher production rate and produce the better-controlled size of NPs. Nonetheless, these methods are considered unfavourable due to high capital cost, high energy requirements and involve the use of toxic and hazardous chemicals. Consequently, these features result in secondary pollution to the environment. Moreover, a previous study demonstrated that the chemical synthesis of NPs is toxic and less biocompatible [29]. Hence, this has limited their clinical and biomedical applications. Therefore, there is a need to explore and develop cleaner, environmentally safe, economical and biocompatible alternatives to synthesize NPs.

In recent years, the green process of NPs has emerged as an alternative to conventional physical and chemical methods by using biological mediated approaches. The biological synthesis of metal and metal oxide NPs involves unicellular and multicellular biological entities including bacteria [30], yeast [14], fungi [31], virus [32] and algae [33]. These methods are cheap, non-toxic and eco-friendly. The microbes act as a tiny nano-factory in reducing the metal ions into metal NPs with the involvement of enzymes and other biomolecule compounds secreted or produced by the microbes. Nevertheless, only a few microbes are reported to have the capability to synthesise ZnO NPs. Hence, there is a need to explore more potential microbes for the synthesis of ZnO NPs. Therefore, the current paper reviews the microbes mediated synthesis of ZnO NPs, the mechanisms of NPs synthesis and optimization parameters and their potential application as an antimicrobial agent and feed supplement in animal industry as well as their toxicological hazards on animal.

### Microbial mediated synthesis of ZnO NPs

NPs have been synthesized by using various conventional physical and chemical methods such as vapour condensation, interferometric lithography, physical fragmentation, sol-gel process, solvent evaporation process and precipitation from microemulsion method [34, 35]. The physical method involves the use of high energy consumption, pressure and temperature, whereas, the chemical method involves the use of perilous and toxic chemicals which contributing in environmental contaminants and hazardous to the person handling it [36]. The toxic chemical that frequently employed in chemical methods is triethyl amine [37], oleic acid [38], thioglycerol [39], and polyvinyl alcohol (PVA) [40] and ethylenediaminetetraacetic acid (EDTA) [41] which is typically used as a capping and stabilizing agent to control the size of NPs and preventing it from agglomeration. Furthermore, some of these hazardous chemicals may reside or bound in the final product of NPs. As such, these may interfere with the biological application as well as limit their usage on animals and human [34]. Collectively, the biological method has gained much interest in the synthesis of metal and metal oxide NPs due to the usage of less toxic chemical, eco-friendly nature and are energy efficient.

The biological synthesis methods of ZnO NPs is performed by using biologically active products from plants and microbes including bacteria, fungi, and yeast. This method is promising owing to its effectiveness, eco-friendly techniques, inexpensive, simple and mass productivity [42]. The biological synthesis using plant extracts is performed using compounds, which are extracted from different parts of the plant such as leaves, roots, stem, fruit and flowers. Some of the plant extracts tend to have complex phytochemical compounds that act as reducing and capping agent in the synthesis process such as phenol, alcohol, terpenes, saponins and protein [43]. Notably, the biological synthesis of metal and metal oxide using plants have been extensively reviewed [34, 44–46]. Hence**,** this paper emphasizes the biological synthesis of ZnO NPs using microbes.

Microbes such as bacteria, fungi, and yeast play an important role in the biological synthesis of metal and metal oxide NPs. In the last decade, the use of microbes has gained increased interest in which there have been many studies conducted using various microorganisms’ models. Nevertheless, the biological synthesis of ZnO NPs using microbes still remains unexplored. Table 1 summarizes several of microbes that mediate the synthesis of ZnO NPs including their size, shape and special applications. Biological synthesis using microbes offers an advantage over plants since microbes are easily reproduced. Nonetheless, there are many drawbacks pertaining to the isolation and screening of potential microbes. The main drawback includes cost-effective of the synthesis processes as it is time-consuming and involves the use of chemical for growth medium. The presence of various enzymes, protein and other biomolecules from microbes plays a vital role in the reduction process of NPs. These multiple organic components secreted in the suspension or growth medium attributed to the formation of multiple sizes, shape with mono- and polydispersed NPs [66]. Moreover, the protein secreted from microbes could act as a capping agent that confers stability of NPs formation.

**Table 1:** Microbes mediated synthesis of zinc oxide nanoparticles

Microbes	Size, nm	Shape	Application	Reference
Bacteria				
*Aeromonas hydrophila*	57.7	Spherical	Antimicrobial	[16]
*Bacillus licheniformis* MTCC9555	250	Flower	Dye removal	[47]
*Bacillus megaterium* (NCIM2326)	45~95	Rod and cubic	Antimicrobial	[15]
*Halomonas elongate* IBRC-M 10214	18.11 ± 8.93	Multiform	Antimicrobial	[48]
*Lactobacillus johnsonii*	4~9	Spherical	–	[49]
*Lactobacillus paracasei* LB3	1179 ± 137	Spherical	Antimicrobial	[50]
*Lactobacillus plantarum* VITES07	7~19	Spherical	–	[51]
*Lactobacillus sporogens*	5~15	Hexagonal	Controlling pollutant	[52]
*Lactobacillus sporogens*	145.7	Hexagonal	Antimicrobial	[53]
*Pseudomonas aeruginosa*	35~80	Spherical	Antioxidant	[54]
*Rhodococcus pyridinivorans* NT2b	100~120	Roughly spherical	UV protection, antibacterial	[30]
*Sphingobacterium thalpophilum*	40	Triangle	Antimicrobial	[55]
*Staphylococcus aureus*	10~50	Acicular	Antimicrobial	[56]
*Streptomyces* sp.	20~50	Spherical	Antimicrobial	[57]
Fungi				
*Alternaria alternata* (Fr.) Keissl (1912)	45~150	Spherical, triangular, hexagonal	–	[58]
*Aspergillus aeneus*	100~140	Spherical	–	[59]
*Aspergillus fumigatus* JCF	60~80	Spherical	Antimicrobial	[60]
*Aspergillus fumigatus* TFR-8	1.2~6.8	Oblate spherical and hexagonal	Agriculture	[61]
*Aspergillus niger*	61 ± 0.65	Spherical	Antimicrobial	[62]
*Aspergillus terreus*	54.8~82.6	Spherical	Antifungal	[63]
*Candida albicans*	25	Quasi-spherical	Synthesis of steroidal pyrazolines	[31]
*Fusarium* spp.	> 100	Triangle	–	[64]
Yeast				
*Pichia kudriavzevii*	10~61	Hexagonal wurtzite	Antimicrobial and antioxidant	[14]
*Pichia fermentas* JA2	n/a	Smooth and elongated	Antimicrobial	[65]

**Fig. 1:**
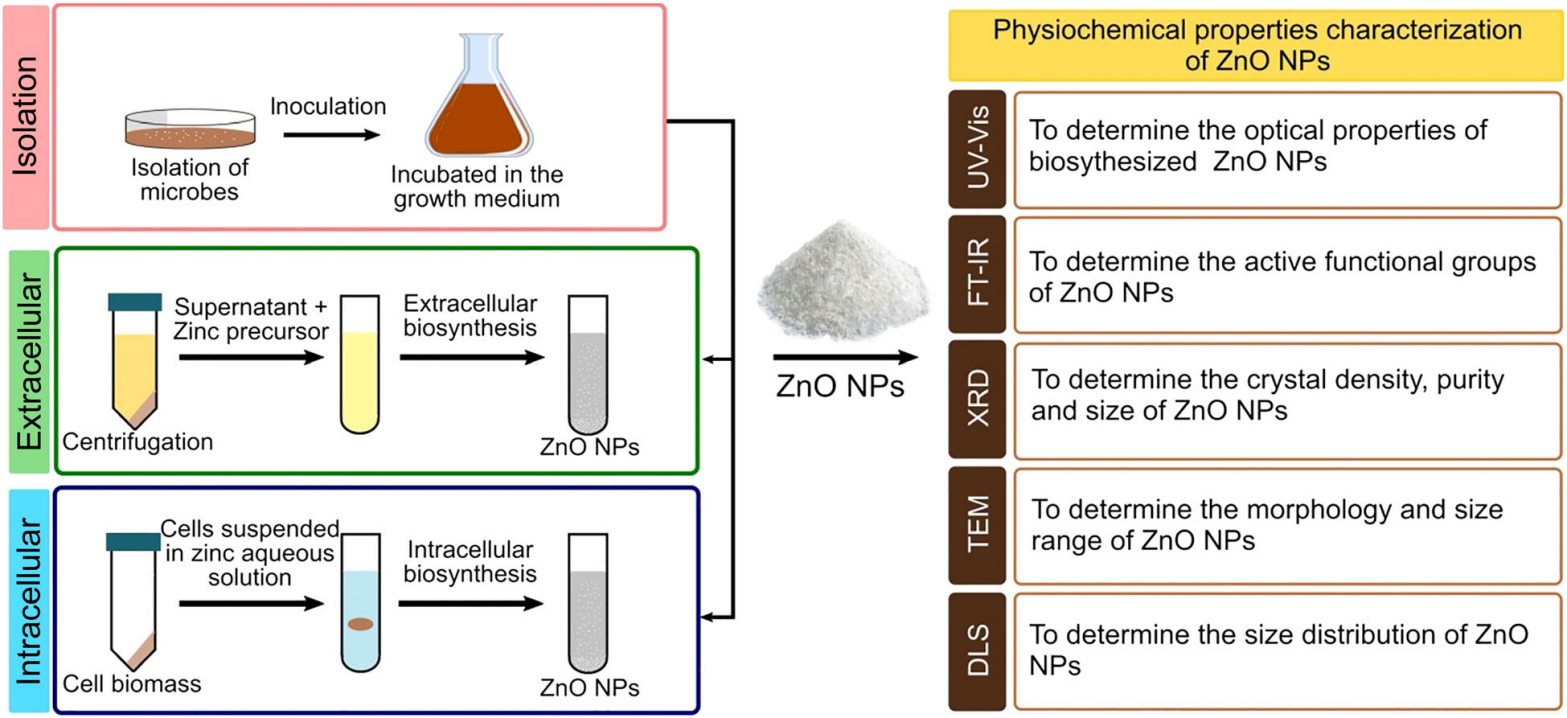
Microbe-mediated synthesis of metal and metal oxide NPs. Microbial synthesis of ZnO NPs requires the selection of microbes, optimal conditions for cell growth, and route of biosynthesis (intra- or extracellular). The ZnO NPs precipitates are washed repeatedly with distilled water followed by ethanol and afterwards dried at 60 °C overnight to obtain a white powder of ZnO NPs. Various physicochemical techniques are used to characterize the properties of NPs, including size, shape, surface charge, functional groups, and purity, by using ultraviolet–visible spectroscopy (UV–Vis), Fourier transform infrared spectroscopy (FTIR), X-ray diffraction (XRD), transmission electron microscopy (TEM), and dynamic light scattering (DLS)

In general, not all microbes are able to synthesize NPs because each microbe has a different metabolic process and enzymes activities. Thus, in this regard, the selection of appropriate microbes (regardless of their enzyme activities and biochemical pathway) is crucial to forming NPs. Generally, the cultures are allowed to grow in the culture medium. Besides, the biological synthesis of metal and metal oxide NPs requires metal precursors, which are usually supplied in the form of soluble salts and precipitated in the suspension containing microbial cells or biological compounds extracts from the microbes. The synthesis reaction is usually completed within minutes or hours depending on the culture conditions, which results in the white deposition in the bottom flasks or changes in the colour of suspensions. Thus, this indicates a successful transformation. Furthermore, several parameters are important to determine the rate of production, yield and morphologies of NPs including the temperature, pH, concentration of metal precursor and reaction time. Figure 1 illustrates the process of a biological method utilizing microbes in the synthesis of metal and metal oxide. The NPs produced are characterized physicochemically to determine their properties including size, shape, surface charge, functional group, and the purity [67].

The synthesis of metal and metal oxide NPs depends on the ability of microbes to tolerate heavy metals. Moreover, it is well-known that high metal stress may have an effect on the various microbial activities [68]. Under stress condition, the microbes tend to reduce ions to respective metals. As such, this demonstrates their capability to act as natural nano-factory [69]. Generally, microbes that inhabit ecological niches rich in metal exhibit high metal resistance due to adsorption of metals and their chelation by intra- and extracellular proteins [70]. Therefore, mimicking the natural biomineralization process could be a promising approach for the synthesis of metal and metal oxide NPs. A number of metal-reducing microbes have been isolated to synthesise the metal NPs. A previous study isolated the soil fungus, *Aspergillus aeneus* from mine in India, which demonstrated a high zinc metal tolerance ability and exhibited the potential for the extracellular synthesis of ZnO NPs [59]. Similarly, another study isolated *Cladosporium oxysporum* AJP03 from the metal-rich soil in India [71]. The fungi were found to have high gold metal tolerance ability and secreted high enzymes and protein. Hence, the fungi was identified as a potential candidate for the extracellular synthesis of gold NPs.

Numerous microbes have been exploited to synthesise ZnO NPs in which bacteria are preferred due to the ease of handling and genetic manipulative attributes compared to other eukaryotic microorganisms [72]. The reproducible bacteria such as lactic acid bacteria (LAB) have attracted increased attention in bacteria mediated synthesis of NPs due to their non-pathogenic properties and high production of various enzymes. Moreover, LAB also recognized as the health beneficial bacteria, which are abundant in the food products [52]. Furthermore, the LAB are facultative anaerobic bacteria that are known to have negative electrokinetic potential. This causes LAB to be easily attracted to the metal ions for the NPs synthesis under both oxidizing and reducing conditions [50, 52]. Apart from that, LAB are Gram-positive bacteria that have a thick cell wall layer consisting of peptidoglycan, teichoic acid, lipoteichoic acid, protein, and polysaccharides [73]. This layer acts as a site for biosorption and bioreduction of metal ions. Additionally, LAB are able to produce exopolysaccharides, which serve as a compound to protect the cell against metal ions and may act as an additional site for biosorption of metal ions [74]. Selvarajan and Mohanasrinivasan [51] demonstrated the intracellular synthesis using *Lactobacillus plantarum* VITES07 that produced a pure crystalline and spherical shape of ZnO NPs with the size ranged from 7 to 19 nm. The authors reported that NPs produced were moderately stable in which the biomolecules secreted by the LAB acted as a capping agent in the synthesis process. Moreover, studies by Mishra et al. [53] and Prasad and Jha [52] demonstrated that using *Lactobacillus sporogens* to synthesis ZnO NPs could produce a similar hexagonal shape with different sizes.

The biological synthesis of ZnO NPs using fungi is a promising approach due to their high tolerance to higher metal concentration, high binding capacity and their ability in metal bioaccumulation over bacteria [75]. Moreover, the fungi exhibited the ability to secrete a large number of extracellular redox proteins and enzymes. As such, this contributed to the reduction of the metal ions into NPs in larger amounts, which is suitable for the large-scale production [66]. The higher amount of protein secreted in the medium by the fungi acted as capping protein that further bound and encapsulated the NPs surface and conferred to the stability. For instance, Raliya and Tarafdar [61] demonstrated the synthesis of ZnO NPs by using *Aspergillus fumigates* TFR-8 that resulted in the formation of NPs with the average diameter size of 3.8 nm and high monodispersity particles (uniformly distributed) without any agglomeration. Moreover, the authors suggested that the protein secreted by the fungi was bound and encapsulated the spherical NPs and prevented the NPs from agglomerate. Subsequently, the stability of NPs was examined for 125 days by measuring the size using particle size analyzer. The results demonstrated that NPs were stable until day 90 and the size increased thereafter due to the agglomeration. This concludes that protein could act as a capping agent to stabilize the NPs up to 90 days. In addition, filtrate-cell free supernatant (FCF) of *Alternaria alternate* was used in the synthesis of ZnO NPs. On that account, the FCF was found to produce NPs after the precipitation of zinc sulfate solution with the size of 75 ± 5 nm. In addition, the FTIR absorption spectra analysis demonstrated the presence of protein and other organic compounds on the ZnO NPs produced. This results corroborated with the previous study that suggested the fungi can generate a high extracellular protein, which bound on the surface of NPs in order to stabilize and prevent it from the aggregation [58]. Therefore, the use of fungi for the synthesis of NPs is favourable as the fungi are efficient in secreting of extracellular enzymes and protein.

Similar to fungi, yeast has been proven to synthesize metallic NPs due to their higher tolerance to the toxic metal. A study conducted by Moghaddam et al., [14] demonstrated that a new isolated *Pichia kudriavzevii* yeast strain was able to synthesize ZnO NPs with ~ 10–61 nm of the size range of NPs produced. The formation of NPs were reported to depend on the reaction duration, which was found to play an important role in the size, shape and distribution of ZnO NPs. Moreover, Chauhan et al. [65] demonstrated an extracellular synthesis of silver NPs and ZnO NPs using *Pichia fermentans* JA2 isolated from the pulp of spoiled fruits. Moreover, the UV-vis spectra results indicated a strong and broad peak at 425 nm and 374 nm implying the successful formation of silver and ZnO NPs, respectively.

The microbes mediated synthesis of ZnO NPs seems to be eco-friendly and safe as it does not involve the use of any toxic and hazardous chemical in the synthesis process. In addition, the biologically active compounds secreted by the microbes were acted as a reducing and capping agents. Thus, this approach is more advantageous than the conventional methods. Furthermore, fungal mediated synthesis seems to be a promising candidate for the synthesis as it produces more biologically active compounds than the other microbes. Nevertheless, in term of the cells growth activity, the bacteria are promising compared to the other alternatives. Moreover, the mechanisms of biological synthesis of ZnO NPs among the microbes are different and are not fully understood yet, hence, further investigation is needed.

### Mechanisms of microbes mediated synthesis of NPs

Evidence has shown that enzymes, protein and other compounds produced by microbes play a vital role in the synthesis process. Nonetheless, to date, the data on the identification of chemical components responsible for the synthesis of NPs has been scant. Microbes exhibit the intrinsic potential to synthesise NPs of inorganic materials, which may be routed either by the intracellular or extracellular pathway. Extracellular synthesis is more advantageous and has been widely applied compared to the intracellular route. This is mainly due to the fact that it could be used to synthesise large quantities and involves simple downstream processing that eliminates various steps of synthesis, easy separation and industrialization. While the recovery process of NPs in the intracellular synthesis requires additional step such as harvesting the cell biomass by centrifugation and subjected to several cycle ultra-sonication for cells disruption to obtain the purified NPs [76]. Nonetheless, the specific mechanism with regard to this has not been completely elucidated.

### Intracellular mechanisms of microbial synthesis

**Fig. 2:**
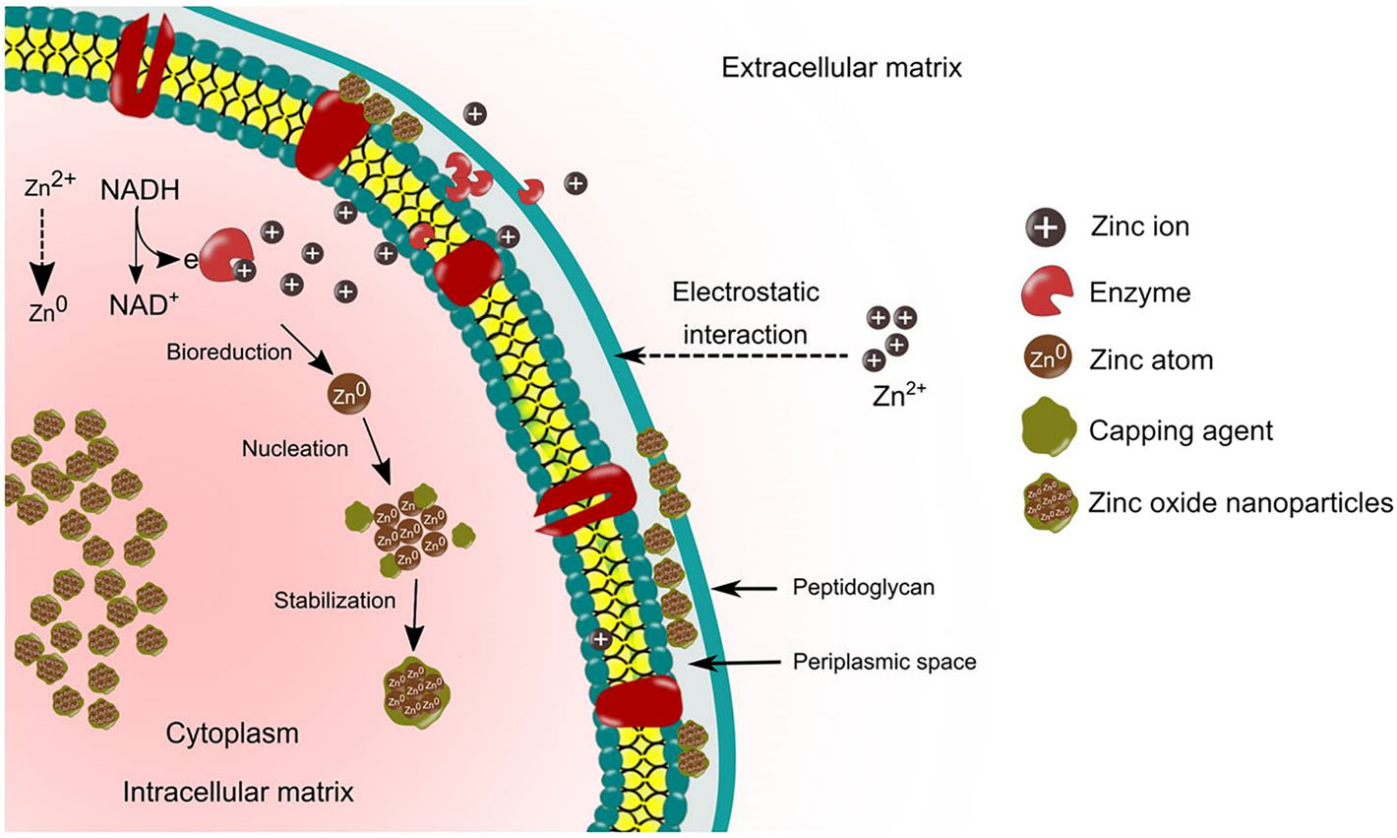
Schematic representation of intracellular synthesis mechanisms of ZnO NPs. The intracellular mechanisms involve the transportation of metal ions into the cell wall by electrostatic attraction. The metal ions are reduced to a metal atom by the enzymes found in the cell wall and then initiate the nuclei growth to form NPs in periplasmic space and cytoplasm. The intracellular synthesis requires ultrasonication to obtain the purified NPs

In the intracellular synthesis pathway, the cell walls of microbes and ions charge play an important role in the synthesis of NPs. This involves distinctive ion transportation in the microbial cell in the presence of enzymes, coenzymes and others. The cell wall of microbes consists of a variety of polysaccharides and protein, which provides active sites for binding of the metal ions [77]. Moreover, not all microbes are able to synthesize metal and metal oxide NPs. Evidence has shown that heavy metal ions exhibit great threat to the microbes in which when there is a threat, the microbes will react by gripping or trapping the ions on the cell wall through the electrostatic interactions [51]. This is due to the fact that metal ion is attracted to the negative charge from the carboxylate groups (specific enzymes, cysteine, polypeptides) that is present on the cell wall [78]**.** Furthermore, the trapped ions are reduced into the elemental atom initiated by the electron transfer from NADH by NADH-dependent reductase that acts as an electron carrier, which is embedded in the plasma membrane. Finally, the nuclei grow to form NPs and accumulate in the cytoplasm or in the cell wall (periplasmic space). On the other hand, the protein or peptides and amino acids such as cysteine, tyrosine and tryptophan exist inside the cells are responsible for providing stabilization of NPs [79, 80]. Figure 2 demonstrates the mechanisms of the microbes mediated intracellular synthesis of ZnO NPs.

Mukherjee et al. [81] demonstrated the intracellular mechanisms using *Verticillium* sp. for the synthesis of NPs involving three steps, which are trapping, bioreduction and capping. The interaction forces between metal ions and the enzymes present on the cell wall reduce the metal ion within the cell wall leading to the aggregation of metal atoms and formation of metal NPs. The authors also reported the presence of metal NPs on the cytoplasmic membrane by transmission electron microscopy (TEM) analysis, which suggested that the formation of NPs occur in both the cell wall and the cytoplasm of the cell. The small metal ions diffused across the cell wall and entered the cytoplasmic membrane. The bioreduction of metal ions into NPs occurred with the presence of local enzymes. Similarly, the intracellular synthesis of gold NPs by utilizing *Rhodococcus* sp. was reported to occur on the cell wall and cytoplasmic membrane that produced 5 to 15 nm size of NPs with better monodispersity form [82].

The intracellular, extracellular route or surface production of NPs have been reported to be pH-dependent. A slower rate of intracellular synthesis of silver NPs synthesis by using *Meyerozyma guilliermondii* KX008616 was observed at pH 3. Moreover, TEM image analysis revealed that small NPs population existed in the cytoplasms in a cluster or nanoaggregates form. Additionally, the NPs were also observed to be located away from the cell wall. The authors suggested that the biomolecules found on the cell such as protein and polysaccharides that are involved in the bioreduction of NPs were inactivated under an extremely acidic condition. Consequently, this caused the metal ion to move into cytoplasms [83]. In another study, the deposition of gold NPs synthesis using *Shewanella alga* cells was reported to depend on the pH conditions. At pH 7, the NPs were deposited in the periplasmic space of the cells whereas, at pH 2, the NPs were observed to be deposited in the cytoplasm and larger NPs were deposited extracellularly (outside the cell) [84]**.** While in other cases, some bacteria species are pH dependent, the membrane-bound oxidoreductases *of L. sporogens* that were used for the synthesis of ZnO NPs were activated under a low pH. This suggests that lower pH ambient is a prerequisite for the synthesis of NPs [52]. Similarly, this was in agreement with a previous study that reported the biological synthesis of ZnO NPs by membrane-bound oxidoreductases of *L. plantarum* VITES07, which was pH-sensitive [51].

### Extracellular mechanisms of microbial synthesis

**Fig. 3:**
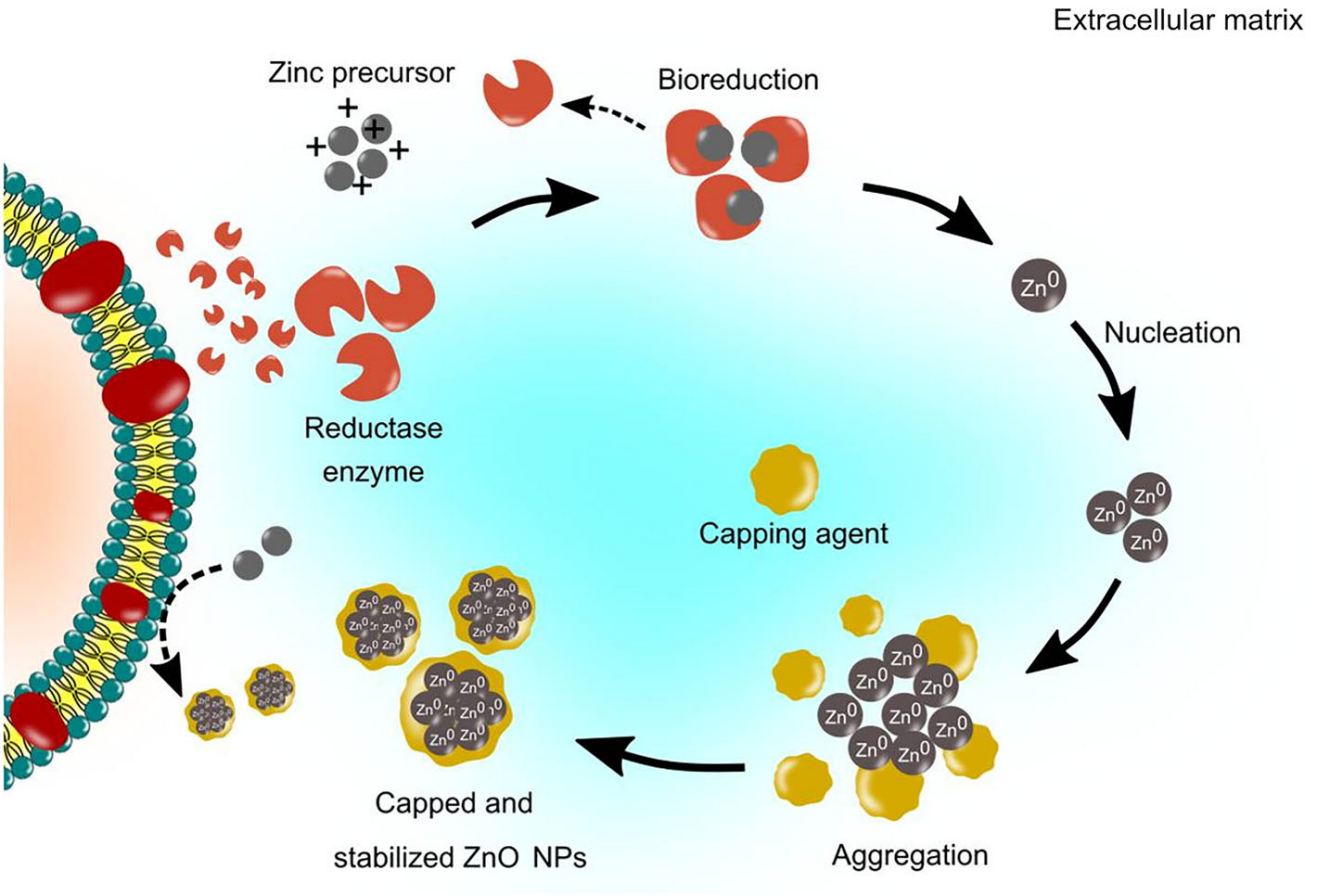
Schematic representation of extracellular synthesis mechanisms of ZnO NPs. The extracellular mechanisms involve enzyme-mediated synthesis such as nitrate reductase enzyme, which is secreted in the growth medium, to reduce the metal ions to their respective metal atoms and lead to nucleation and growth of NPs. The extracellular protein secreted by the microbes acts as a capping agent for NPs stabilization. The formation of white precipitation in the medium shows the production of NPs

Numerous studies reported that extracellular synthesis is a nitrate reductase-mediated synthesis, which is responsible for the reduction of metal ions into metal NPs [30, 65, 85–87]. The extracellular synthesis pathway involves enzyme-mediated synthesis which located on the cell membrane or the releasing of the enzyme to the growth medium as an extracellular enzyme. Nitrate reductase is an enzyme in the nitrogen cycle that catalyses the conversion of nitrate to nitrite. For instance, the bioreduction of Zn^2+^ was initiated by the electron transfer from NADH by NADH-dependent reductase that acts as an electron carrier [88]. Consequently, the Zn^2+^ obtained electron and reduced to Zn^0^. Subsequently, this resulted in the formation of ZnO NPs. The schematic of the extracellular synthesis mechanisms is illustrated in Fig. 3.

Kundu et al. [30] conducted an experiment to determine the involvement of the secreted protein or enzyme by the multi-metal tolerant bacteria, *Rhodococcus pyridinivorans* NT2 for the synthesis of ZnO NPs. The bacteria biomass were exposed to zinc ion and millipore water as a control. The extracellular protein from the supernatant was determined by a protein expression profile. The results of Lowry’s assay demonstrated that the concentration of extracellular protein secreted by the bacteria biomass that was exposed to zinc ions was twice higher than in the control, which was 1113 ± 6.3 μg/mL and 554 ± 7.1 μg/mL for control. Moreover, the electrophoretic profile by one-dimensional SDS-PAGE revealed that the presence of a molecular mass of 43 kDa, which was an NADH-dependent reductase. The findings indicated that NADH-dependent reductase resulted in the formation of ZnO NPs.

Studies have shown that protein produced and secreted by microbes play an important role in the NPs synthesis [57, 89–91]. Nevertheless, some studies suggested that the native form of the protein is not compulsory for the NPs synthesis process. A study by Jain et al., [59] revealed that amino acids present in the protein were found to interact with the Zn^2+^ ions to form NPs. The study also investigated the ability of denatured (heat treated) and native (untreated) protein present in the fungal cell-free filtrate suspension for the synthesis of ZnO NPs. The results demonstrated that both heats treated and untreated samples were able to synthesize ZnO NPs. Notably, the absorbance spectra result by UV-Vis demonstrated a higher reaction rate on the heat treated protein compared to the untreated. This indicates that the synthesis of ZnO NPs was higher in the heat-treated samples. Hence, the results confirmed that the presence of the native form of the protein is not mandatory for the synthesis process. This may attribute to the fact that, the interaction between hydrogen bond and non-polar hydrophobic was disrupted during the heating process. Consequently, this resulted in the exposed contact of amino acids with zinc ions that led to the formation of ZnO NPs [59]. Moreover, the authors also suggested that the biosynthesis of metal NPs was non-enzymatic due to the denaturation of the structure of the enzyme during heat treatment.

In some cases, the non-enzymatic mediated synthesis depends on the certain organic functional groups present on the microbial cell wall, which facilitates the reduction of metal ions. The live cell biomass and dead cell (heat killed by autoclaving) of *Corynebacterium glutamicum* were used to synthesize silver NPs. After sonication of the cell, the UV-Vis spectra results demonstrated a strong plasmon resonance between 400 and 450 nm for both samples. Both samples were further incubated for a few days. The results indicated that the peak area and height of the UV-Vis spectrum for the dead cell were comparatively higher compared to live cell samples. This indicates a higher productivity of silver NPs [92]. The authors also validated the results by investigating the total organic content (TOC) for both samples and revealed that the TOC in dead cells was twice higher than the live cell. This indicates the release of organic molecules (reducing agent) from the cell due to rupturing of the cell walls during the heat-killed in which the silver ions obtained the access to more organic molecules and hence a higher amount of reduction occurred [92]. Therefore, the study confirmed that the formation of NPs could occur without the involvement of biological enzymes or metabolites compounds. Nevertheless, this was in contrast to Korbekandi et al. [74], which suggested that the NPs synthesis was an enzymatic reaction. The study demonstrated that the boiling of the *Lactobacillus casei* biomass killed the bacterial cells and denatured the enzymes. The results demonstrated the presence of NPs in the reaction mixture with active biomass, whereas, no absorbance was observed in the reaction mixture with boiled biomass. This indicates that the enzymes found in the medium were denatured while the dead cells were unable to secrete enzymes resulting in absence of synthesis of NPs. Thus, it can be speculated that the inconsistent finding was due to variations in the methods and species of microbes used for the synthesis of NPs.

The protein secreted by microbes could also act as a capping agent despite acting as a reducing agent. As such, this facilitated the higher stabilization and dispersion of NPs [30]. As such, several studies demonstrated the involvement of protein as a capping agent. For instance, Velmurugan et al., [64] investigated the role of protein in live, dried and dead biomass of *Fusarium* spp. as a capping agent in the synthesis of ZnO NPs. The SEM-EDS results on the NPs produced by live and dried biomass demonstrated signals of Na and K. This indicated the bound of proteins on the surface of zinc crystallites. This result was supported by the FTIR results, which demonstrated clear peaks. The findings revealed the presence of protein and amide, I and II bands at 1100, 1400, 1650, 2900 and 3000 cm^− 1^, respectively. Nevertheless, there was no protein signal detected in zinc crystal produced by the dead biomass. Similarly, Bao et al. [93] evaluated the chemical composition of the ligands capping on the NPs. The FTIR results demonstrated two absorption bands at 1650 and 1566 cm^− 1^, which indicated the typical amide I and II absorptions of protein molecules, respectively. Further verification was carried out via protein purification by using high-performance liquid chromatography (HPLC) to analyze the molecular mass of the capped protein. The results demonstrated the presence of two proteins with molecular mass of 7.7 kDa and 692 kDa. Additionally, the authors suggested that the yeast used in the experiment facilitated the synthesis of NPs and generated protein ligands to act as a capping agent, which inhibited the aggregation of NPs.

### Effect of various parameters on the optimization process of NPs synthesis

**Fig. 4:**
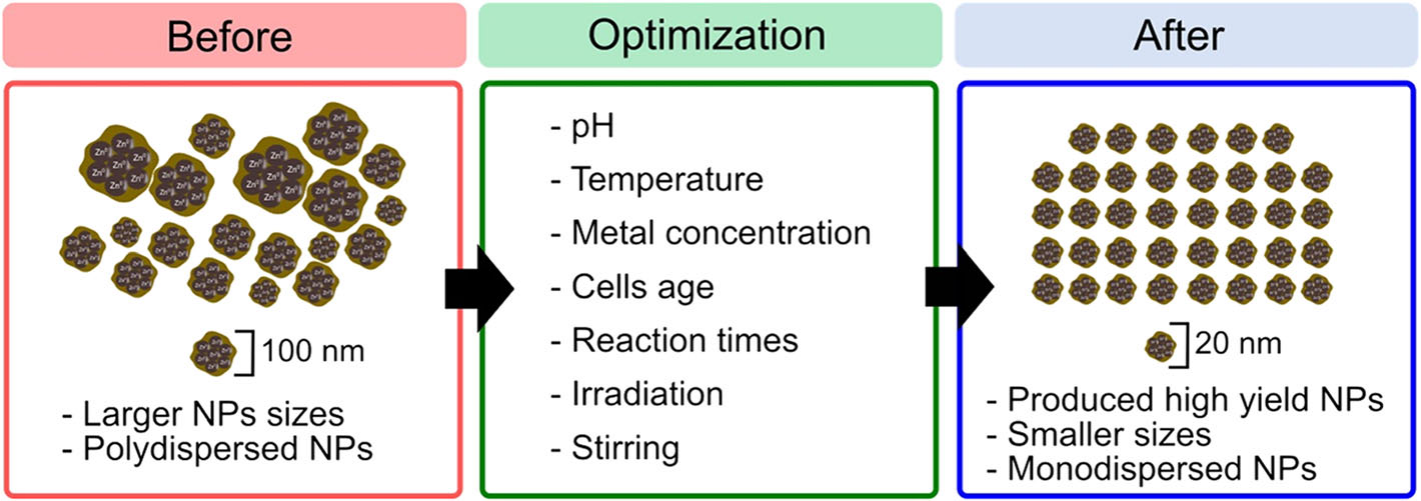
Strategies for optimizing the synthesis of ZnO NPs. The synthesis of NPs is associated with different physicochemical parameters including pH, temperature, precursor concentration, microbe age, reaction times, irradiation, and stirring. Each of these parameters contributes to variations in size, shape, monodispersity, and yield of NPs

Microbes-mediated synthesis of NPs has the potential to be a great alternative to chemical and physical methods, despite the main drawback in applying biological synthesis of NPs, which refers to the difficulty in controlling both the size and the shape of NPs. The main major concerns in using microbes are to increase yield production for industrial scale, which demand further investigation. It has been widely reckoned that the physicochemical properties of NPs are highly dependent on their size and morphology structures. Studies have proven the direct effects of NPs size and shape on their performance. Sadeghi et al., [94] revealed that the nano-plate-shaped NPs exhibited good antibacterial activity due to their large surface area, in comparison to those with nano-rod shape. In another study, ZnO NPs at 12 nm effectively inhibited the growth of pathogenic bacteria, when compared to those at 212 nm [95]. Therefore, in order to generate effective size distribution, morphologies, and yield production of NPs, it is necessary to optimize both the cultural condition and the varied physical parameters, including pH, temperature, metal ions concentration, microbial age, and reaction time. The biological synthesis of NPs seems to gain better commercial acceptance if the NPs are produced in high yield with the desired size and shape. The schematic representation of parameters for producing the desired NPs is portrayed in Fig. 4.

### Effect of pH

Generally, pH is a key factor that has a major role in the synthesis of metal NPs, mainly because pH has the ability to alter the shape of biomolecules that is responsible in capping and stabilizing the NPs [96]. Gericke and Pinches [97] assessed the biosynthesis of gold NPs using *Verticillium luteoalbum* by varying the pH level to determine its impact on the size and shape of the generated NPs. The outcomes displayed a majority smaller size of NPs with spherical shape obtained at pH 3, in comparison to those retrieved for pH values 7 and 9 that predominantly produced larger NPs with irregular and undefined shapes. Optimization of pH may also be influenced by the species of microbes applied in the synthesis. For instance, an acidophilic bacterium, *Lactobacillus casei*, resulted in increased absorbance of silver NPs production as the pH value was reduced; indicating that growth and enzymes activity of *L. casei* are better in weak acidic environment [74]. In the case of alkaline condition, Gurunathan et al., [98] asserted that hydroxide ion is essential to decrease metal ions. The authors observed rapid increment in silver conversion within less than 30 min’ reaction time at pH 10; signifying that the protein, which served as a reducing agent, was present in the supernatant and was active in reducing power under alkaline conditions. Additionally, the authors confirmed their results by observing the NPs with TEM analysis that recorded smaller size of NPs ranging between 10 and 15 nm. The finding is in agreement with that reported by Ma et al., [99], which recorded increase in absorbance peak of silver NPs with increment of pH value. The synthesis of silver NPs using *Fusarium oxysporum* at pH 6 resulted in the smallest size, whereas higher pH generated the biggest size, which indicated the catalytic activity of enzymes involved in the synthesis of NPs that appeared to be deactivated under alkaline condition, thus causing an increase in the size of NPs [29].

### Effect of temperatures

Numerous researches have investigated the impacts of various temperatures on the size and yield production of NPs. Mohammed Fayaz et al., [100] assessed the effects of temperatures on NPs size produced by *Trichoderma viride* at 10 °C, 27 °C, and 40 °C. The UV-Vis spectra outcomes showed that lower wavelength regions at 405 nm were obtained at 40 °C and higher wavelength regions at 420 nm and 451 nm were obtained at 27 °C and 10 °C, respectively, indicating increment in NPs size at higher wavelength regions. The author also verified their results with TEM analysis that showed a high temperature of 40 °C generated smaller monodisperse NPs size ranging between 2 and 4 nm, while at a lower temperature, larger NPs were produced. In another study, maximum production of silver NPs synthesized by *Sclerotinia sclerotiorum* was obtained at 80 °C with 10–15 nm size range. This postulated that higher temperature increased the kinetic energy, thus leading to rapid synthesis rate and maximum NPs with a smaller size [101]. The decrease in particle size with increased temperature is normally due to increment in reaction rate at higher temperature. This causes the metal ion to be consumed rapidly in forming nuclei, while the size is reduced initially due to reduction in the aggregation of the growing NPs [98].

### Effect of precursor concentration

The impact of various precursor salt concentrations on the synthesis of metal NPs using soil fungus *Cladosporium oxysporum* revealed that the optimum concentration of precursor salt at 1.0 × 10^− 3^ mol/L gave maximum NPs yield. Nonetheless, at concentrations 2.0 × 10^− 3^ and 5.0 × 10^− 3^ mol/L, no NPs was generated due to the insufficient biomolecules in minimizing the high amount of metal ions present [71]. This finding is in agreement with that reported by Jamdagni et al. [12], who discovered that absorbance of ZnO NPs by UV-Vis spectra increased with increment of precursor concentration (2.5 × 10^− 5^ to 1.0 × 10^− 4^ mol/L). They added that further increment in concentration (2.0 × 10^− 4^ mol/L) resulted in broad peak, while decrease in absorbance signified reduction in the synthesis of ZnO NPs. The influence of metal ion concentration on the synthesis of silver NPs using *Penicillium aculeatum* Su1 suggested that high concentration of metal ions increased the aggregation of NPs, which resulted in the formation of larger NPs size. The authors reported that maximum production of NPs yield was obtained at absorbance peak of 415 nm by UV-vis spectra, whereby as the concentration increased to 2.5 × 10^− 3^ mol/L, the absorbance peak shifted to 435 nm; signifying the increased size of NPs formation [99]. Meanwhile, another study reported that increment in metal ions concentration to a certain point generated NPs with smaller size. The study of silver NPs synthesis by using extracellular supernatant of *Escherichia coli* revealed that increment in silver ion concentration up to 5 × 10^− 3^ mol/L minimized the size of NPs by about 15 nm, in which the authors speculated that the silver ions bound on the growing particles to form a coat that prevented them from aggregation [98].

### Effects of microbial age and reaction time

The growth phase of cell is essential for the synthesis of NPs. Since microbes generate various enzymes at different growth phases, controlling the cell age may be useful in producing high yield of NPs. Gericke and Pinches [97] reported that the biomass of *Verticillium luteoalbum* harvested at 24 h produced a high yield of gold NPs, when compared to biomass harvested at 72 h. This may be attributed to the fact that cell at the early exponential stage actively generated high concentrations of enzymes and protein, which resulted in high reduction of metal NPs. In a study pertaining to ZnO NPs synthesis that employed *Pichia kudriavzevii*, prolonged reaction time was discovered upon exposure to metal ions at 36 h that produced aggregate with irregular-shaped NPs, whereas reaction time of 12 and 24 h generated the smallest size of NPs [14]. On the other hand, ZnO NPs synthesis that employed *Lactobacillus* sp. yielded NPs with an average size of 7 nm for 5 to 10 min of reaction time [51].

In summary, microbes have been reckoned to generate NPs. Nevertheless, optimization process is essentially required to produce the desired NPs size, shape, yield, and homogeneous particles (monodispersity), mainly because these NPs have a significant role in determining their unique properties for specific applications. The study is still ongoing because each microbe has a wide range of abilities in producing NPs and further investigation is required to improve the synthesis process for implementation in practice.

### The potential application of ZnO NPs in animal industry

**Fig. 5:**
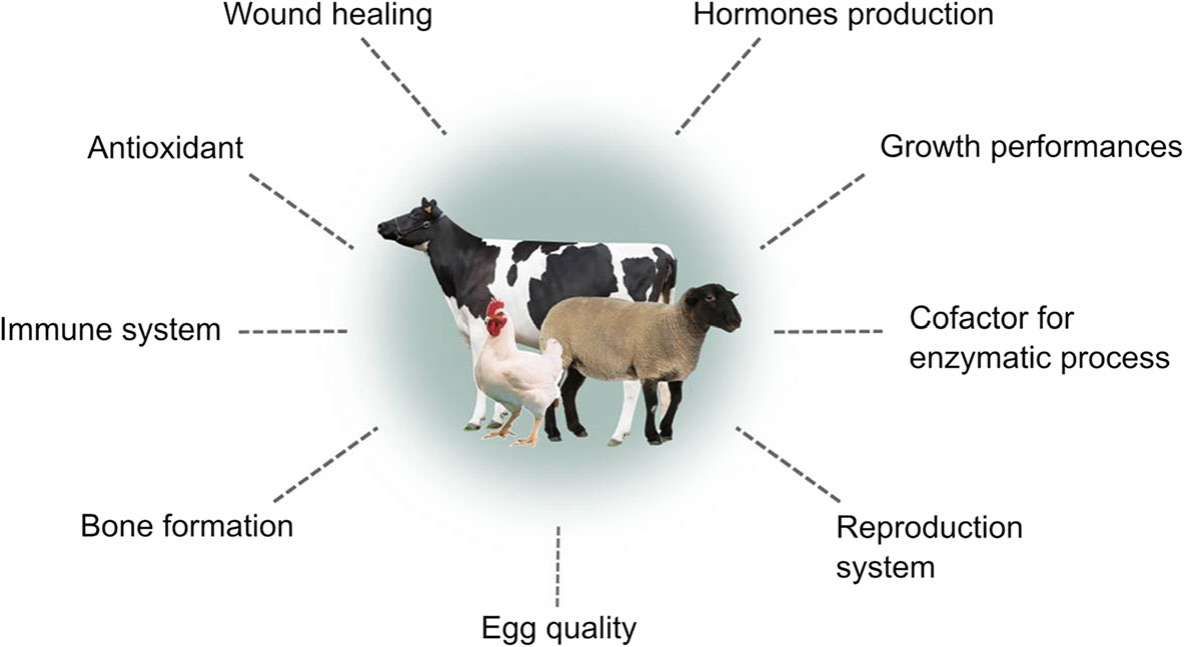
Role of zinc supplementation in poultry and livestock. Zinc is an important trace element for physiological and biological functions of the body. The utilization rate of zinc in the animal’s body is low and therefore the addition of ZnO NPs to animal feeds is believed to increase zinc uptake and bioavailability in the body

ZnO NPs is one of the largest produced metals oxide [2] which has been extensively studied due to its unique properties of semiconductor characterized by broad direct band gap width (3.37 eV) with high excitation binding energy (60 meV) and deep borderline ultraviolet (UV) absorption [102]. In this regard, its unique and attractive properties have made it a promising tool for application in many industrial areas including pharmaceutical [103], cosmetic [104], photocatalyst [102], UV light emitting devices [105] and agriculture industries [9, 106]. Zinc plays a significantly important role in a variety of physiological processes in human, animals as well as plants. It is extensively present in all body tissues including muscles, bones, and skin [107], In addition, zinc is an integral component in numerous enzyme structures [11] and has a crucial part in hormone secretion, growth, reproduction, body immune system, antioxidant defence system and many other biochemical processes in the body [17]. Figure 5 illustrates the role of zinc in poultry and livestock.

ZnO NPs possess many valuable features including their eco-friendly materials, biocompatibility, biodegradability and most importantly their bio-safety traits which have been graded by the US Food and Drug Administration [4]. Furthermore**,** ZnO NPs have been found to exhibit non-toxic properties in human cells at a certain concentration level [108]. In fact, biological mediated synthesis of ZnO NPs does not involve any hazardous chemical and material, thus making their application in living organisms safe. The efficiency of ZnO NPs is greater than their counterparts due to their high surface to volume ratios. Therefore, the use of bulk zinc oxide has been widely replaced with ZnO NPs in many aforementioned applications. With the onset of biological mediated synthesis of NPs, the application of ZnO NPs has extended into the next level of application particularly in the field of biomedical and nutrition in human and animals. In the recent year, ZnO NPs have been extensively investigated for use in animal husbandry and production as an antimicrobial agent for disease prevention and as a feed supplement in animals diet to improve the utilization efficiency of trace elements in the animal’s body.

### Potential role as an antimicrobial agent in animal industry

The continuous usage of conventional antibiotic has led to the growth and spread of multidrug-resistant strains [109]. Thus, the discovery and development of new approaches as an alternative to a conventional antibiotic is necessary. ZnO NPs produced by the biological enzymatic process have varied application and have been prominently studied recently on their excellent antimicrobial activities such as antibacterial [13] and antifungal [12]. The distinctive features of NPs such as their small size in relation to a large surface area, composition and morphology allow the NPs to interact with the bacterial cell surface and penetrate the cell’s core and subsequently exhibit bactericidal mechanisms [110]. Moreover, the inorganic antibacterial properties of NPs’ materials have the ability to withstand extremely harsh conditions and high temperatures compared to organic materials [111]. Microbes mediated synthesis of ZnO NPs could become potential antimicrobial agents as some of the bacterial species are able to produce a variety of compounds that exhibit antimicrobial properties which are known as bacteriocin. Bacteriocin is a small heat-stable peptide which has a bactericidal effect on pathogenic microorganisms [112]. The bacteriocin derived from the microbes could act as a reducing agent for the synthesis of metal NPs [113]. In addition, previous study has proved that this small peptide could also bind to the surface of NPs as a capping agent which in turn enhance the antimicrobial effects of metal NPs [112–116]. Nonetheless, there is a lack of study on employing the biological synthesized of ZnO NPs by microbes on animal production which possibly due to their limitation on mass production for large scale application. Furthermore, many *in vitro* studies have been conducted on the antibacterial ability of ZnO NPs [14–16, 67], however, the exact mechanism of antibacterial activity of ZnO NPs remains elusive.

### Antibacterial mechanisms of ZnO NPs

Scientists have suggested a few possible bactericidal mechanisms, some proposed that smaller NPs have greater surface reactivity and easier cell penetration that released the Zn^2+^. The release of Zn^2+^ from ZnO NPs is one of the main propositions in antibacterial mechanisms which are known to inhibit several bacterial cells activities including active transport, bacteria metabolism and enzymes activity. Subsequently, the toxicity properties of Zn^2+^ on the bacterial cell biomolecules induced the cell to death [117]. Moreover, the release of Zn^2+^ is size and morphology dependent. For instance, the release of Zn^2+^ in smaller size spherical structures of NPs is higher than in rod structures due to its smaller surface causing equilibrium solubility [118]. While the other proposed antibacterial activity is caused by the formation of reactive oxygen species (ROS) which leads to oxidative stress and subsequent cell damage or death. The formation of ROS is a common antibacterial activity adopted by ZnO NPs [34] which are generated under UV exposure and consist mainly of reactive species such as superoxide anion (O_2_^−^), hydroxyl ion (OH^−^) and hydrogen peroxide (H_2_O_2_). These reactive species are generated from the surface of the NPs that react with the hydroxyl groups and absorb water (H_2_O) to create hydroxyl radicals (OH^−^) and H^+^ and consequently creates a superoxide anion (O_2_^−^) with the presence of O_2_ [67]. The O_2_^−^ will then react with H^+^ to produce HO_2_ and generate into H_2_O_2_ in the presence of electrons and H^+^ [13]. Eventually, the H_2_O_2_ penetrates the bacterial membrane and damage the cellular components such as lipid, protein and DNA resulting in injuries and cells death [119]**.** However, OH^−^ and O_2_^−^ are unable to enter the membrane of bacteria cell due to their negative charge and may be found on the outer surface except for H_2_O_2_ [111].

**Fig. 6:**
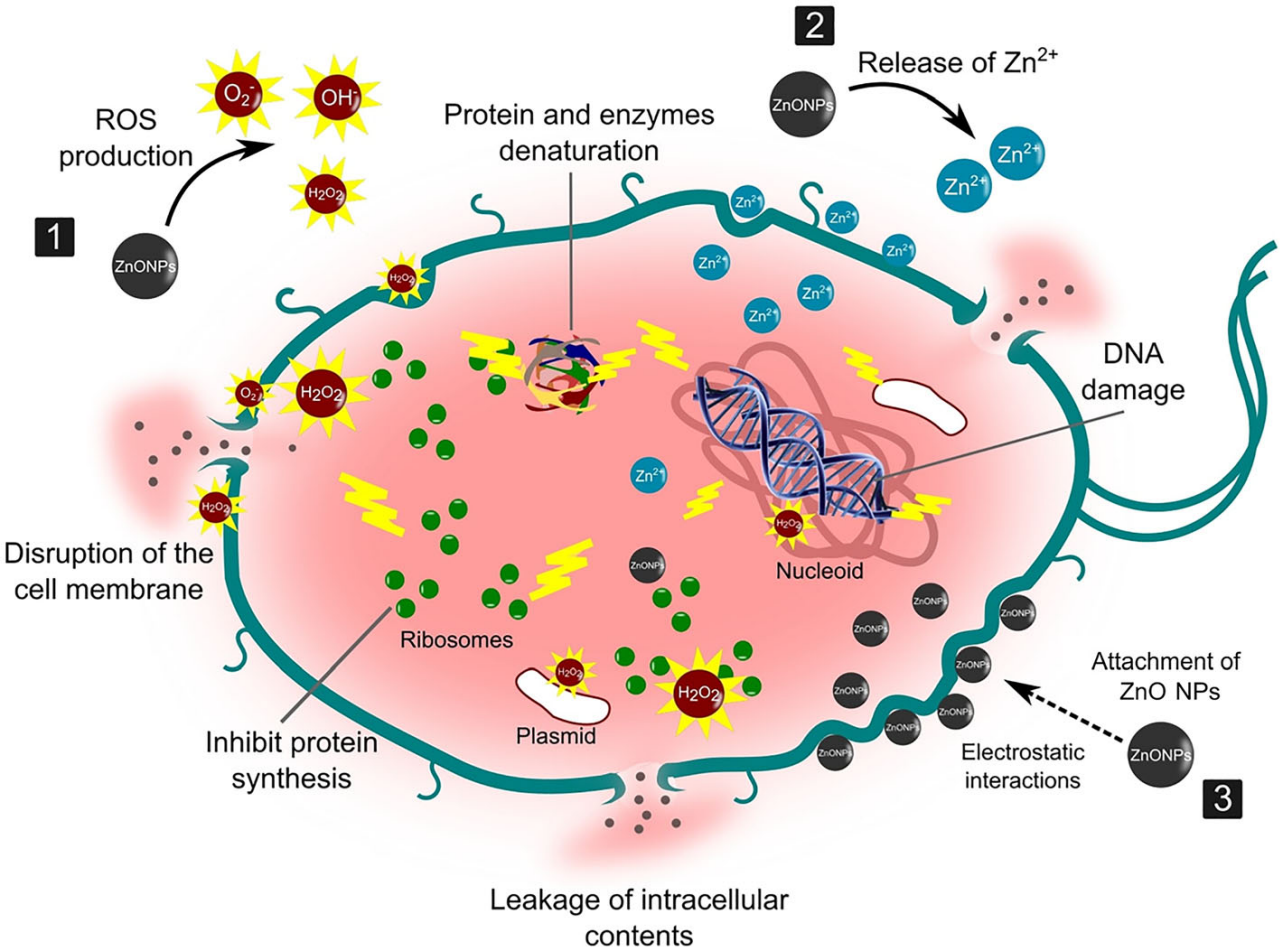
Schematic illustration of the antimicrobial mechanism of ZnO NPs against bacterial cells. ZnO NPs act as an antimicrobial agent through the following mechanisms: (1) the formation of reactive oxygen species (ROS), which induces oxidative stress and membrane and DNA damage, resulting in bacterial death; (2) dissolution of ZnO NPs into Zn^2+^, which interferes with enzyme, amino acid, and protein metabolisms in bacterial cells; and (3) direct interaction between ZnO NPs and cell membrane through electrostatic forces that damages the membrane plasma and causes intracellular content leaks

Another possible mechanism for the antimicrobial activity of ZnO NPs is through the attachment of NPs to the bacteria cell membrane via electrostatic forces. The positive zeta potential of ZnO NPs promotes the attachment to the negatively charged bacterial cell which leads to the penetration of ZnO NPs into the cells [110]. This interaction may distort the membrane plasma structure and damage the bacterial cell integrity, resulting in the leakage of intracellular contents and ends with cell death [16]. In addition, the accumulation of ZnO NPs in the cell also interfered with the metabolic functions of the bacteria that leads to death. The mechanism of ZnO NPs antibacterial activity is illustrated in Fig. 6. Therefore, the aforesaid bactericidal mechanisms provide better action modes compared to the conventional therapeutic agents tendency to develop multidrug-resistant microorganism.

### Antimicrobial activity of ZnO NPs in animal industry

In the poultry and livestock industry, *Salmonella* and *Campylobacter* are common enteric foodborne pathogens which can be found in the gut and skin of the animals [120, 121]. These pathogenic bacteria can be transmitted from animals to human through the handling of animals and the consumption of contaminated undercooked meat and egg products [122]. *Staphylococcus aureus* a pathogenic bacteria present in meat product cause food poisoning and is also responsible for bovine mastitis and bumblefoot disease in poultry [123, 124]. Furthermore, *S. aureus* has the ability to build a resistance rapidly with the prolonged use of antibiotics [125]. *Escherichia coli* is another pathogenic bacteria commonly colonized in human and domestic animal gut microbiota. In poultry, *E. coli* is the major factor of mortality in newly hatched young chicks [126] which contribute to economic losses in the poultry industry. Therefore, NPs has arisen to be a new approach in the reduction of these pathogenic bacteria colonization in animals without the risk of developing multi-drug resistance.

The well-diffusion test using a biologically synthesized of ZnO NPs against various Gram-positive and Gram-negative bacteria and fungus was carried out to evaluate their antimicrobial activity. The results showed a maximum zone of inhibition was observed in *Pseudomonas aeruginosa* and *Aspergillus flavus,* 22 ± 1.8 mm and 19 ± 1.0 mm respectively [16]. Similarly, the extracellular synthesis of ZnO NPs employing the endophytic bacteria *Sphingobacterium thalpophilum* showed antimicrobial effects against *P. aeruginosa* [55]. In other studies, the synergistic effects experiment were carried out by combining the ZnO NPs with various antibiotics including tigecycline, vancomycin, erythromycin and ofloxacin to test their ability in various multidrug-resistant bacteria. The results showed an increase in antibacterial activities in the presence of ZnO NPs among the resistant bacteria and effective zone of inhibition was observed in *Enterococcus* sp., *Staphylococcus aureus* and *Proteus mirabilis* [65]**.** While marine yeast mediated synthesis of ZnO NPs was found to have an effective antibacterial activity against the human pathogens of *E. coli* and *B. subtilis.* The well-diffusion test showed a larger inhibition zone against *E.coli* and *B. subtilis* at a concentration of 100 μg/mL. However, the antibacterial activity of ZnO NPs was more efficient against *E. coli* compared to *B. subtilis* due to their different cell wall composition for Gram-negative and Gram-positive [57].

Furthermore, the bactericidal activity of ZnO NPs is dependent upon their size, shape, stability and concentration. A green mediated synthesis of rectangular shaped ZnO NPs employing stevia leaves demonstrate an effective bactericidal activity against *S. aureus* and *E. coli.* The results showed the minimum inhibitory concentration (MIC) value was 2.0 μg/mL and that the rectangular shaped ZnO NPs had a higher antimicrobial effect at lower concentration [127]. In one comparative study on the antibacterial effect of ZnO NPs against their bulk particle counterparts, ZnO NPs was found to exhibit higher toxicity effects on *B. subtilis, E. coli* and *P. fluorescens*, while their counterparts had none or lower toxicity. This indicates particles size do make a difference in toxicity [128]. The results showed NPs size exhibit greater antibacterial activities over their counterparts’ size.

A nano-rod shaped ZnO NPs produced by *Bacillus megaterium* cell-free supernatant was tested for its antibacterial activity against multidrug-resistant *Helicobacter pylori* strain. The TEM analysis revealed cells exposed to ZnO NPs at a concentration of 17 μg/mL for 60 min showed a disruption of cell membrane causing leakage of the cellular content which induced the cell to death. Whereas the cell unexposed to ZnO NPs remained complete with intact cell membrane. The authors also suggested that the nano-rods shaped NPs acted like a needle penetrating the bacterial wall and damaging the cells [15].

Mycotoxin is a common contaminant in animal feed produced by fungi-producing mycotoxins such as *Aspergillus, Penicillium* and *Fusarium* genera [129]. A high percentage of mycotoxins contamination in animal feed has been reported [130] with adverse effects seen in the animals’ performance and health. ZnO NPs were tested for their antifungal potential. The ZnO NPs synthesis by *Aeromonas hydrophila* showed a maximum inhibition zone of antifungal activity against *A. flavus* (19 mm ±1.0 mm) [16]. Moreover, Jamdagni et al., [12] conducted an antifungal test of biological mediated synthesis of ZnO NPs against *Alternaria alternata*, *Aspergillus niger*, *Botrytis cinerea*, *Fusarium oxysporum* and *Penicillium expansum* found ZnO NPs were effective against all the tested fungi and *A. niger* was found to be sensitive to ZnO NPs with the lowest MIC value of 16 μg/mL. Thus, from this point of view, ZnO NPs could become a potential antifungal agent substitute for conventional fungicides and possibly prevent the development of fungicides resistance.

Furthermore, one of the most common enteric diseases in poultry and livestock farming is coccidiosis. Coccidiosis is a protozoan disease caused by enteric protozoa of the genus *Eimeria* infecting the intestinal mucosa and resulting in bloody diarrhea, reduced weight gain and high mortality in poultry and livestock farming [131]. ZnO NPs have also been reported to exhibit anticoccidial properties. Dkhil et al., [132] in an *in vivo* study on the properties of ZnO NPs anticoccidial activity in mice infected with *Eimeria papillata* showed that mice infected with *E. papillata* produced 29.7 × 10^3^ ± 1500 oocysts/g of feces compared to the treated infected mice decreased excretion of 12.5 × 10^3^ ± 1000 oocysts/g of feces.

Despite the excellent antimicrobial ability of ZnO NPs against pathogenic microorganisms, their usage as an antimicrobial agent in animal husbandry remains underutilized due to the lack of proposed strategies for *in vivo* assessment. However, several studies have demonstrated the effects of ZnO NPs supplementation on the gut microbiota of domestic animals [133, 134]. In an intestinal microbiome study by Yausheva et al. [134], the supplementation of ZnO NPs in broiler chicken resulted in the highest biological activity on cecal microbiota and the authors recommended ZnO NPs to be considered as a potential bactericidal drug for broiler chicken. In another study, hens fed with ZnO NPs for 9 weeks showed a decrease of bacterial richness in the ileum, particularly *Lactobacillus*; however, the effect observed was dose-dependent [133]. The reduction of *Lactobacillus* in the gastrointestinal tract is of particular interest because it is a predominant genus in the animal gut [135] that plays a significant role in regulating the level of some pathogenic bacteria [136]. Nonetheless, Yausheva et al. [134] found that even though the supplementation of ZnO NPs resulted in the decrease in the number of *Lactobacillus*, there was no increase in the level of pathogenic microorganisms in the cecum of the broiler chicken. This indicates that the supplementation of ZnO NPs is also able to regulate pathogenic microorganism in the animal’s intestinal tract. This is contradictory to the study by Xia et al. [137], which reported an increase in microbiota richness and diversity in the ileum and colon of piglets fed with 600 mg/kg of ZnO NPs. Furthermore, the authors also noted the increase in the abundance of Firmicutes, Lactobacillaceae, and *Lactobacillus* in the colon, which may be beneficial and contribute to a more stable gut microecosystem. Similarly, Milani et al. [138] observed stabilization of gut microbiota on weaned piglets fed with ZnO NPs at concentrations of 15, 30, and 60 mg/kg. To summarize, the intrinsic properties of ZnO NPs make them an ideal antimicrobial agent substitute for conventional antibiotics. Moreover, the specific mechanism of ZnO NPs is different from conventional antibiotics, hence preventing the development of multidrug-resistant bacteria. In addition, numerous *in vitro* studies on their antimicrobial activity have proven that ZnO NPs exhibit remarkable capability in inhibiting the growth of a wide spectrum of bacterial species at possibly lower doses. Nonetheless, the main concern in applying ZnO NPs as the potential antimicrobial agent in animal husbandry is their destructive effects on the beneficial microorganisms of the intestinal microbiota. Hence, more investigation is needed to elucidate their effects on the gut ecosystem of animals.

### ZnO NPs as a dietary supplement in animal industry

Zinc is an essential micronutrient component for animals. It influences their body’s physiological and biological functions including growth, reproduction, wound healing, body immune system, DNA, protein synthesis, oxygen free radical scavenging and as a component of numerous enzymes in animals [9]. The zinc bioavailability in the animal’s body is low [139], thus regular dietary intake is required. Generally, the forms of zinc sources used in animal feed are inorganic zinc such as zinc oxide (ZnO) and zinc sulfate (ZnSO_4_) and organic zinc such as zinc propionate, zinc methionine, and zinc acetate [140, 141]. The organic zinc is higher in bioavailability compared to inorganic zinc, but the application of organic zinc in animal diets is limited due to its high cost [140, 142]. Moreover, the biggest issue in using conventional inorganic zinc as a feed supplement in the animal’s diet is their low utilization rate, hence, animal feed manufacturers and producers used a greater amount of dietary zinc than the recommended normal requirement to achieve maximum performance of the animals [143]. The excessive addition of zinc in the feed subsequently lead to excess zinc in the excreta which causes adverse effects on the environment [144]**.** Apart from that, the high dietary supplement of zinc may also affect the stability of vitamins and other nutrients in the animal’s body [142].

**Fig. 7:**
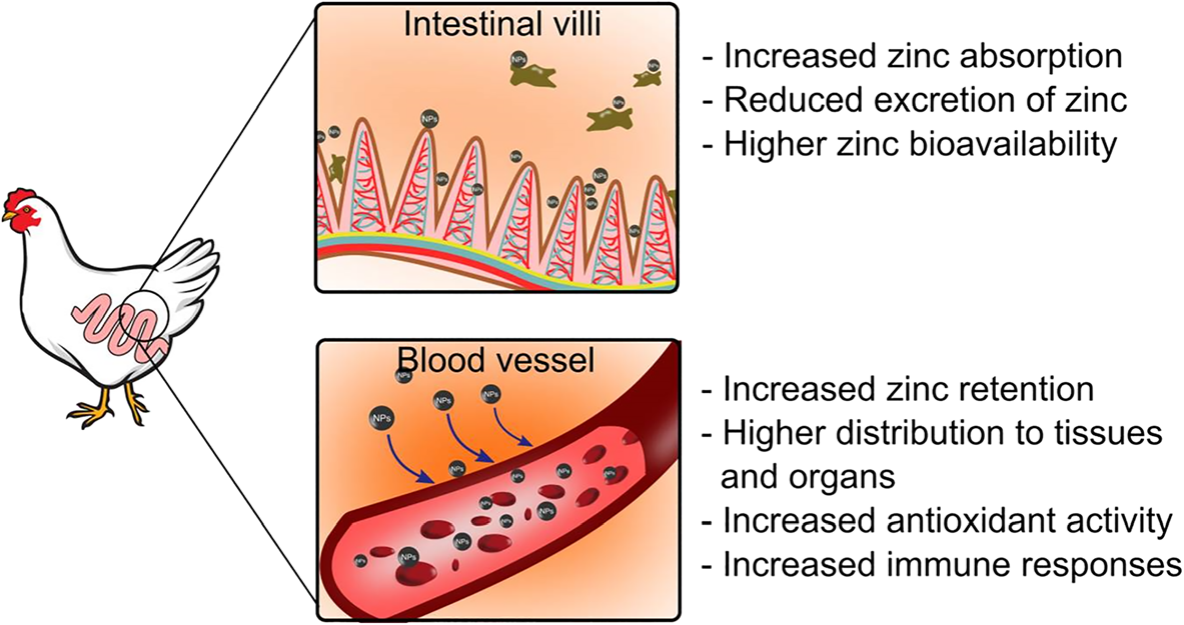
Distribution of ZnO NPs in animal body. ZnO NPs has the capability to cross the gastrointestinal tract and then further distribute into the blood and into the targeted organs

The emergence of nanotechnology associated with nanoscale has improved the bioavailability and utilization efficiency of trace elements in animal’s diets [145]. Recently, ZnO NPs have been prominently studied on their effects in animal production and their potential application as a dietary supplement as an alternative to the conventional zinc [22, 142, 146, 147]. Due to their small size, ZnO NPs have been introduced into animal feed to increase and improve the absorption rate of zinc in the gastrointestinal tract, which would increase the uptake of zinc and bioavailability in the animal’s body [19, 24]**.** High bioavailability of ZnO NPs also reduces the secretion of zinc in feces hence alleviate the environmental pollution. Apart from that, the small size of NPs would easily cross into the blood and distribute the NPs to the internal organs (Fig. 7).

The use of ZnO NPs in animal feed has been reported to enhance the growth performance of the animals. Broiler chicken was fed with diets containing 20 and 60 mg/kg of ZnO NPs and showed an increase in body weight gain and better feed conversion ratio compared to the use of conventional zinc [142]**.** However, the higher concentration of ZnO NPs (100 mg/kg) inhibits the growth performance which indicates that the effects of ZnO NPs supplementation depend on their level of concentration and should be fed at the appropriate level [142]. In another study, the dietary supplement of ZnO NPs at 60 and 90 mg/kg had significantly improved broiler carcass yield by increasing the live body weight, dressing and carcass weight [20]**.** Generally, about 2000 to 4000 mg/kg of dietary zinc supplement is added into the feed in weaning piglets in order to promote the growth performance and to prevent the occurrence of diarrhea [148]**.** This inclusion of excessive zinc is higher than the recommended normal requirement and therefore will increase the feed cost and cause an environmental impact. Wang et al. [22] demonstrated a feeding trial on weaned piglet using ZnO NPs and conventional ZnO plus colistin sulfate at 1200 mg/kg and 3000 mg/kg, respectively. The results showed that weaned piglets supplemented with ZnO NPs had improved growth performances with alleviating diarrhea and the interestingly similar result was also reported in dietary treatment with conventional ZnO at higher doses. Therefore, the use of ZnO NPs in lower doses was found to be efficacious and can be substituted for the higher doses of conventional ZnO and also prevent the excretion of excessive zinc in feces into the environment.

The thin eggshell is a common problem in old layer hens which resulted in easily broken eggs. The thickness and strength of eggshell were found to be enhanced in dietary supplemented with ZnO NPs and Zn-methionine compared to the conventional ZnO. The inclusion of ZnO NPs in the diets also showed the highest egg production [25]. This may be attributed to the fact that the important role of zinc in the synthesis and secretion of the reproductive hormone which contributed to the high bioavailability and uptake efficiency of ZnO NPs for the production of eggs. In addition, the use of ZnO NPs in the study had increased the thickness of the eggshell and was found similar to the results of dietary with Zn-methionine (organic zinc) [25]. Zinc is known as a component of the carbonic anhydrase enzyme which plays an important role in the formation process of eggshell as well as in improving the strength of the eggshell [28]. In another study, the thickness of the eggshell was also found improved in layer hens supplemented with organic zinc and ZnO NPs, contributed by the high bioavailability and zinc retention in the body [24]. Therefore, supplementation of ZnO NPs in layer hens could enhance the egg’s quality and solve the problem of thin eggshells in the old layers and the efficacy of ZnO NPs was found higher as organic zinc and could substitute the use of organic zinc.

In recent years, several studies have used a combination of probiotics mixed with nano minerals including selenium NPs [149] and ZnO NPs [147]. It has been suggested that probiotic has the ability to decrease the pH of intestine due to the modulation of gut microflora resulting in the increased of short-chain fatty acids [150] which increase the mineral solubility and absorbability [151]**.** A synergistic effect was observed in the dietary combination of probiotic and ZnO NPs on the improvement of villus height and width of broiler chicken. The results showed a better improvement of villi height to crypt depth ratio in broilers fed with ZnO NPs (50 mg/kg) and probiotic compared to conventional zinc oxide and ZnO NPs (25 mg/kg) [147]. Intestinal morphology is important for better absorption of nutrients as larger villi enable intestine greater absorption of nutrients. Supplementation of probiotic together with ZnO NPs has significantly improved the intestine morphology thus provide a better absorption of other nutrients and increases the body’s health. A dietary supplementation of ZnO NPs alone also has greater effects on the improvement of intestinal morphology. A weaned piglet was fed a basal diet supplemented with 1200 mg/kg ZnO NPs showed increases in villus width, length and surface area which will provide greater absorption ability [22].

Zinc is an essential mineral which acts as a catalyst or coenzyme factors in many enzymes including superoxide dismutase (SOD). SOD acts as an essential component in the antioxidant defence system which plays a significant role in the detoxification of superoxide free radicals and protects the cells against oxidative stress [152]. Fathi et al., [27] fed 20 mg/kg of ZnO NPs to broiler chicken and showed a significant effect on copper-zinc-superoxide dismutase (Cu-Zn-SOD) activity. Cu-Zn-SOD is a metalloenzyme which belongs to the ubiquitous family of SOD [153]. However, no significant effect was observed on the Cu-Zn-SOD activity at higher concentration. In addition, greater SOD enzyme activity was found in the liver and pancreas tissue of layer hens when supplemented with ZnO NPs at 80 mg/kg and organic zinc compared to the conventional zinc [25]**.** It has been known that the bioavailability of ZnO NPs and organic zinc is high thus lead to greater zinc retention and lower the excretion as well as increased the activity of SOD. Furthermore, catalase is also known as an antioxidant enzyme which functions to protect the cells from oxidative damage by ROS [154]. The decrease in catalase activity is related to the increase in oxidative stress [155]. In Zhao et al. [142] study, catalase activity in serum was significantly higher in broiler chicken fed with 20 mg/kg of ZnO NPs which indicated supplementation of ZnO NPs induced the antioxidant activity. However, higher supplementations of ZnO NPs at 100 mg/kg eventually inhibit the catalase activity in the liver tissue samples.

Zinc supplementation provides a better immune system function. Zinc is essential for thymulin to produce peripheral T-cell and thymocytes through the thymus secretion [156]. Thus, the high bioavailability of zinc contributes to the increase of thymulin activity and therefore, promotes the immune responses in the animal’s body. The inclusion of ZnO NPs at 80 mg/kg into the diet was found to increase the sheep red blood cells (SRBC) antibody titre in layer hens compared to the conventional zinc. The higher cellular immune response to antibody titres against the Newcastle disease was also found in the diet with ZnO NPs [25]. In addition, a comparative study of dietary supplementation between inorganic (conventional inorganic zinc), organic (zinc-methionine) and ZnO NPs on broiler chicken found higher antibody titres against SRBC in the dietary treatment of organic and ZnO NPs compared to the conventional zinc [18]. This result indicates that the bioavailability of zinc in the body plays a significant role in a better immune response. In summary, zinc is one of the important trace elements in an animal body for biological functions. Numerous studies have verified the efficiency of ZnO NPs over conventional zinc, and what is more, the efficacy of ZnO NPs is better as organic zinc. Moreover, ZnO NPs can be used as an alternative to the conventional zinc which will reduce the quantity required.

### Toxicological effects of ZnO NPs on animals

Despite their potential use as a feed supplement, ZnO NPs also tend to cause adverse effects on animals. However, the toxicological hazards of ZnO NPs remain controversial because while a few studies have reported ZnO NPs to have therapeutic benefits, other studies reported their toxicity on living organisms. Nevertheless, studies have suggested that the toxicity effects of ZnO NPs are dependent on their concentration (dose) [157], size [158, 159], morphology, and surface composition [160]. Moreover, the toxicity mechanisms of ZnO NPs still remain unclear. However, it has been proposed that they can easily enter cells or bind with the membrane or release Zn^2+^ and generate oxidative stress-mediated DNA damage and lipid peroxidation, which subsequently cause apoptosis [9, 67, 144].

Several studies have reported that high doses of ZnO NPs supplementation could lead to toxicity [161–164]. The results of an *in vivo* experiment conducted by Wang et al. [162] show that the supplementation of high doses ZnO NPs at 5000 mg/kg caused toxicity in mice by decreasing their body’s weight and increasing the relative weight of the pancreas, brain, and lung. Moreover, zinc accumulation was also observed in the liver, pancreas, kidney, and bones. Meanwhile, long-term exposure to ZnO NPs at 50 and 500 mg/kg only showed minimal toxicity. Furthermore, oral administration of ZnO NPs (20 mg/kg body weight) in lambs caused toxicity effects, which were increased levels of blood urea nitrogen (BUN) and creatinine, indicating renal dysfunction [163]. In the histopathological examination, a high concentration of oral administration of ZnO NPs at 400 mg/kg induced focal hemorrhages and necrosis on the liver and heart tissue of Wistar rats, which were caused by oxidative stress [165]. In another study, Wang et al. [22] carried out serum biochemical assay to determine the toxicity of lower dosage of ZnO NPs supplementation on weaned piglets. Enzymes such as glutamic oxaloacetic transaminase (GOT), glutamic-pyruvic transaminase (GPT), and lactate dehydrogenase (LDH) are important biological parameters to evaluate the possible toxicity *in vivo*. There were no effects in serum activity (GOT, GPT, LDH) in weaned piglets fed with 1200 mg/kg ZnO NPs, indicating that supplementation of ZnO NPs at a certain level of concentration does not lead to toxicity [22].

The toxicity effects of NPs are also associated with their sizes and shapes. The smaller NPs (3–6 nm) are more easily cleared out from the kidneys compared to bigger NPs (approximately 30 nm), which remain in the liver [166]. Furthermore, bigger NPs also tend to stay longer in the kidneys due to the slower excretion mechanisms of glomerular filtration and this long-term retention can lead to organ toxicity [167]. In addition, different morphologies of NPs also contribute to the toxicity effects regardless of their specific surface area. Wahab et al. [168] investigated the cytotoxicity effects of ZnO NPs with different morphologies such as nanoplates, nanorods, nanosheet, and nanoflower on malignant human T98G gliomas and fibroblast cells. Nanorods demonstrated higher cytotoxicity and inhibitory effects on cancer and normal cells, respectively, due to a larger effective surface area that potentially induces higher oxidative stress on cells. Moreover, all aforesaid studies used chemically synthesized ZnO NPs, which could be one of the possible causes of the innate toxicity of NPs due to the chemical reaction conditions in the conventional method.

Due to the accumulated scientific reports on the toxic nature of ZnO NPs, several strategies have been employed to produce safer NPs without affecting their unique physicochemical properties. Surface-bound chemical modification is a commonly used method to alter the surface of NPs which play a crucial role in their biological interactions [169]. Using this method, NPs are coated with selective substances such as silica [170, 171], organosilanes [172], chitosan [173], and polyethylene glycol [169]. Chia et al. [170] used a thin silica coating for surface modification of ZnO NPs, which was effective in reducing their cytotoxicity effect on epithelial cells by restricting the dissociation of ZnO NPs to Zn^2+^. However, the silica coating is not ultimately benign because high concentrations of silica-coated NPs still induced cytotoxicity to mammalian gut cells [170]. Among the coating substances, polyethylene glycol (PEG) is widely used for surface modification of NPs due to its biocompatibility and biodegradation properties [67]. Several studies have reported that PEG coating is very effective in inhibiting the toxicity of NPs by modulating the release of Zn^2+^ and ROS production [174]. PEG-coated ZnO NPs was reported to have reduced cytotoxicity on human acute leukemia cell line (THP-1) [169]. Furthermore, Martinez et al. [174] carried out a study to compare the cytotoxicity effect of PEG-coated ZnO NPs and uncoated ZnO NPs on breast cancer MCF-7 cell line. Cells treated with PEG-coated ZnO NPs had higher viability, which indicates that surface modification with PEG interferes with the pathways of cytotoxicity, while the uncoated ZnO NPs shows cytotoxicity on the tested cell line. Surface modification approach is potentially a great strategy in reducing the toxic hazards of NPs; however, this strategy needs to be reconsidered when considering its manufacturing cost for large-scale production. In summary, the toxicity effects of ZnO NPs are caused by their dosage, size, and shape; thus, the use of ZnO NPs in animal diets should be restricted to a specific minimum concentration to avoid their toxic effects. Moreover, for improved safety of ZnO NPs, microbe-mediated synthesis should be considered in NPs production due to its biocompatibility as well as controllable NPs size and shape which can be achieved through the optimization process.

## Conclusions and future prospects

The biologically active compounds secreted by the microbes have dual role functional groups in reducing and stabilizing agent. The microbial synthesis process is easier, simpler and does not involve any hazardous chemicals. Nevertheless, there remain challenges in the microbes mediated synthesis to obtain the desired NPs and to produce high yield NPs. Thus, many optimization processes have been performed by varying the physicochemical parameters and the type of microbes to obtain desirable NPs as well as increase the yield, however, further investigation is needed to understand the formation mechanisms of NPs due to variation between different microbes’ species.

ZnO NPs contain promising properties to be implemented in the poultry and livestock industry. Moreover, ZnO NPs exhibit potential use as the therapeutic agents due to their bactericidal effects on the wide spectrum of bacteria and fungi. As such, this can potentially replace the conventional antibiotics that tend to develop multidrug-resistant bacteria. Furthermore, the use of ZnO NPs as the feed supplement in the animal diet revealed a better bioavailability and high absorption rate due to to their smaller size compared to conventional inorganic zinc sources. The use of ZnO NPs increased the utilization rate of zinc in the body and alleviated the environmental impact by reducing the amount of zinc in the diet and thus decreased the undigested zinc in excreta.

Studies have shown that the conventional chemical and physical methods may potentially contain toxic chemical bound on the surfaces of NPs, which may cause the toxicity effects in the body. Despite having the potential application as an antimicrobial agent and nutrient component, ZnO NPs also tend to cause toxicity in the body. The toxicity effects could be caused by their size, shape and concentration of NPs. Nevertheless, to date, there is no data available on the use of biological mediated synthesis of ZnO NPs to be tested on the poultry and livestock feed. Thus, further investigation is needed to evaluate the effects of ZnO NPs that are produced using microbes to be used as a feed supplement in the diet as well as the antimicrobial agents. Moreover, microbes mediated synthesis have the ability to control and manipulate the size, shape and produce desireable NPs which would offer the advantage over chemical and physical methods. In addition, the source of ZnO NPs from microbial synthesis may have additional properties on their functional groups. This may enhance their chemical and biological function, where it is safe to be used in the living organisms. Furthermore, there is a need to explore more potential microbes in the synthesis of ZnO NPs as there are only a few potential microbes that are reported in the literature. There are diverse species of microbes in the environment; hence, it is necessary to screen novel groups of microbes. In addition, the biochemical and molecular mechanisms with regard to the synthesis mechanisms by microbes should be further elucidated. Therefore, biological synthesis of ZnO NPs by utilizing microbes could represent a new source of key advancements in sustainable agriculture, particularly in the animal industry.

## Data Availability

Data sharing is not applicable to this article as no datasets were generated or analysed during the current study.

## Abbreviations

DLS: Dynamic light scattering
FTIR: Fourier-transform infrared spectroscopy
LAB: Lactic acid bacteria
NADH: Nicotinamide adenine dinucleotide
ROS: Reaction oxygen species
SDS-PAGE: Sodium dodecyl sulphate-polyacrylamide gel electrophoresis
SEM: Scanning electron microscopy
SOD: Superoxide dismutase
TEM: Transmission electron microscopy
UV-Vis: Ultraviolet-visible spectroscopy
ZnO NPs: Zinc oxide nanoparticles
